# Reducing deposition of encrustation in ureteric stents by changing the stent architecture: A microfluidic-based investigation

**DOI:** 10.1063/1.5059370

**Published:** 2019-01-04

**Authors:** A. Mosayyebi, D. Lange, Q. Yann Yue, B. K. Somani, X. Zhang, C. Manes, D. Carugo

**Affiliations:** 1 Department of Mechanical Engineering, Faculty of Engineering and Physical Sciences, University of Southampton, Southampton SO17 1BJ, United Kingdom; 2 Institute for Life Sciences (IfLS), University of Southampton, Southampton SO17 1BJ, United Kingdom; 3 Department of Urologic Sciences, Faculty of Medicine, University of British Columbia, Vancouver V6H 3Y8, Canada; 4 Department of Urology, University Hospital Southampton NHS Trust, Southampton SO16 6YD, United Kingdom; 5 Department of Environment, Land and Infrastructure Engineering, Politecnico di Torino, Turin 10129, Italy

## Abstract

Ureteric stents are clinically deployed to retain ureteral patency in the presence of an obstruction of the ureter lumen. Despite the fact that multiple stent designs have been researched in recent years, encrustation and biofilm-associated infections remain significant complications of ureteral stenting, potentially leading to the functional failure of the stent. It has been suggested that “inactive” side-holes of stents may act as anchoring sites for encrusting crystals, as they are associated with low wall shear stress (WSS) levels. Obstruction of side-holes due to encrustation is particularly detrimental to the function of the stent, since holes provide a path for urine to by-pass the occlusion. Therefore, there is an unmet need to develop novel stents to reduce deposition of encrusting particles at side-holes. In this study, we employed a stent-on-chip microfluidic model of the stented and occluded ureter to investigate the effect of stent architecture on WSS distribution and encrustation over its surface. Variations in the stent geometry encompassed (i) the wall thickness and (ii) the shape of side-holes. Stent thickness was varied in the range 0.3-0.7 mm, while streamlined side-holes of triangular shape were evaluated (with a vertex angle in the range 45°-120°). Reducing the thickness of the stent increased WSS and thus reduced the encrustation rate at side-holes. A further improvement in performance was achieved by using side-holes with a triangular shape; notably, a 45° vertex angle showed superior performance compared to other angles investigated, resulting in a significant increase in WSS within “inactive” side-holes. In conclusion, combining the optimal stent thickness (0.3 mm) and hole vertex angle (45°) resulted in a ∼90% reduction in encrustation rate within side-holes, compared to a standard design. If translated to a full-scale ureteric stent, this optimised architecture has the potential for significantly increasing the stent lifetime while reducing clinical complications.

## I. INTRODUCTION

Ureteric stents are clinically deployed to retain ureteral patency and adequately decompress the kidney, by maintaining ureteral flow in the presence of an intrinsic or extrinsic obstruction.^1–3^ The most commonly used stent (known as “double-J”) consists of a hollow polymeric tube residing within the ureter lumen, with curly ends at its extremities to achieve stable anchoring within both kidneys and the bladder. Circular side-holes are often manufactured along the body of the stent, to provide a pathway for urine to by-pass the obstruction.^4^

Since the introduction of the first stent by Finney in 1978,^5^ a large body of work has been carried out to redesign stents to improve their overall performance.^6^ Despite these efforts, encrustation and biofilm-associated infections remain significant complications of ureteral stenting,^7^ potentially leading to the functional failure of the stent. They are also responsible for increased patient morbidity and healthcare costs, as they often require hospital re-admission and re-intervention.^8^

While efforts to prevent stent-associated complications have primarily focused on varying the surface properties or bulk material of the stent,^9–11^ only few studies have considered altering the stent architecture to enhance urine drainage, improve passage of stone fragments, or reduce adhesion of bacterial and encrusting particles. For instance, Finney introduced a grooved design with the aim of facilitating passage of stone fragments post-lithotripsy, by enhancing bulk urine flow.^12,13^ Similarly, Anderson and Maerzke^14^ designed a helical stent that was believed to improve urine drainage by expanding the ureteral lumen. Although this design demonstrated an increase in urine flow compared to the more traditional double-J architecture,^15^ it still suffered from both bacterial adhesion and encrustation.^16^ Tong *et al.*^17^ utilised a computational model to evaluate a stent with extruded side-holes, in order to increase inter-compartmental flow exchange between intra- and extra-luminal compartments of the stent. While promising, this design is complex to manufacture, and the extruded side-holes may cause tissue irritation upon deployment thereby increasing patient discomfort.

Earlier experimental and theoretical studies have postulated that increasing urine drainage within the stented ureter may result in lower encrustation rates.^18,19^ However, a limitation of the designs reported above is that they were not guided by a quantitative correlation between relevant flow metrics and the formation of microbial or crystalline deposits. This has hindered the identification of critical regions of the stent that are prone to failure and that would merit further design optimisation efforts.

In recent years, microfluidic-based devices have proven to be an effective tool to capture the formation of crystals and probe their physical properties under finely controlled fluidic environments. For instance, microfluidic platforms integrated with optical and spectroscopic instrumentation have been employed to determine the physical structure and chemical composition of pharmaceutical salts,^20^ biominerals,^21^ salts in saline aquifers,^22^ and pathological microcalcifications.^23^ In our previous study, we developed “stent-on-chip” (SoC) models replicating key hydrodynamic regions of the stented and occluded ureter.^23,24^ Using optical microscopy and numerical simulations, we revealed an inverse correlation between wall shear stress (WSS) and deposition of encrusting particles over the stent surface. Moreover, we identified regions of the stent suffering from low-WSS, including “inactive” side-holes (i.e., holes that do not actively contribute to urine drainage) and the hydrodynamic cavity formed by a ureteric obstruction. These findings are coherent with observations on stents retrieved from patients, where the majority of side-holes were plugged with crystals.^25^

Building on our previous findings, in this study we utilised SoC models as an investigational platform to evaluate the effect of changing the stent thickness and shape of side-holes, with the aim of identifying stent architectures capable of reducing crystal deposition and overall stent encrustation.

## II. MATERIALS AND METHODS

### A. Stent-on-chip (SoC) models: Design rationale

**FIG. 1.**
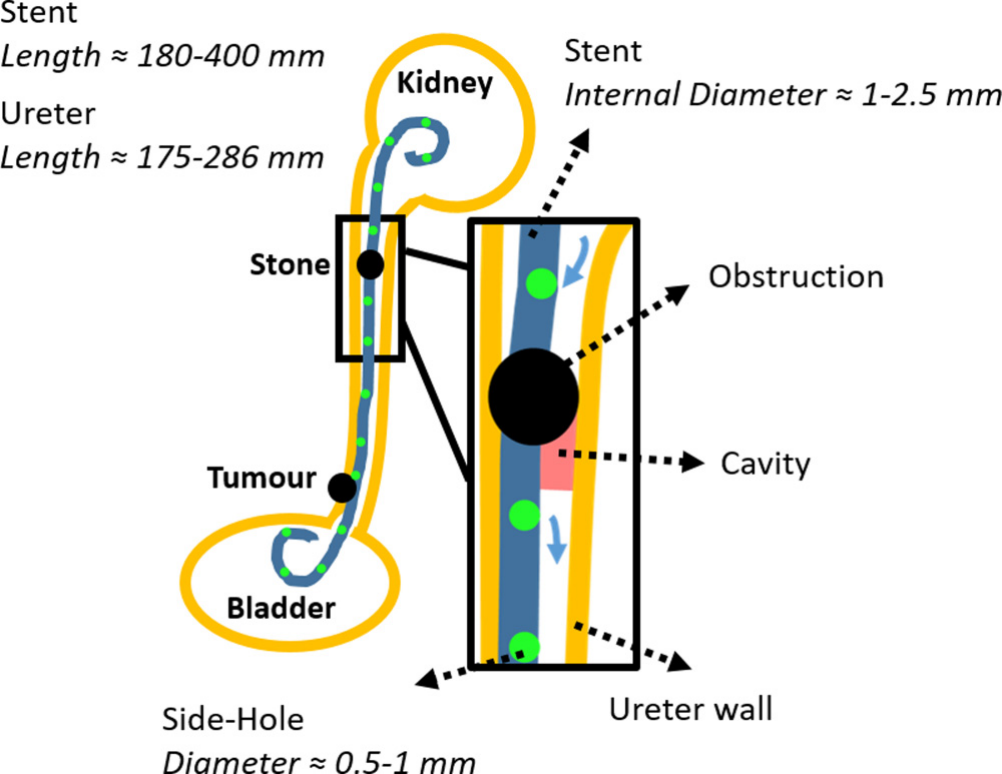
Two-dimensional (2D) schematic of the urinary system, including a simplified representation of ureteric obstructions (intrinsic and extrinsic) and a double-J ureteric stent. Side-holes of the stent are illustrated as green circles. A zoomed-in view of the region close to an intrinsic obstruction of the upper ureter is shown in. The hydrodynamic cavity generated by a complete occlusion of the upper ureter lumen is also shown in red, and blue arrows indicate inter-compartmental urine flow at side-holes near the occlusion. Typical values for relevant geometrical characteristics of both ureter and stent are also reported (the stent's length comprises its pigtail ends).

The SoC model employed in this study was designed to replicate some characteristic hydrodynamic features of a stented and occluded ureter, as described in detail in our previous work.^24^ A schematic of the clinical problem is illustrated in Fig. 1, with indicated relevant dimensions and the hydrodynamic regions modeled in this study.

**FIG. 2.**
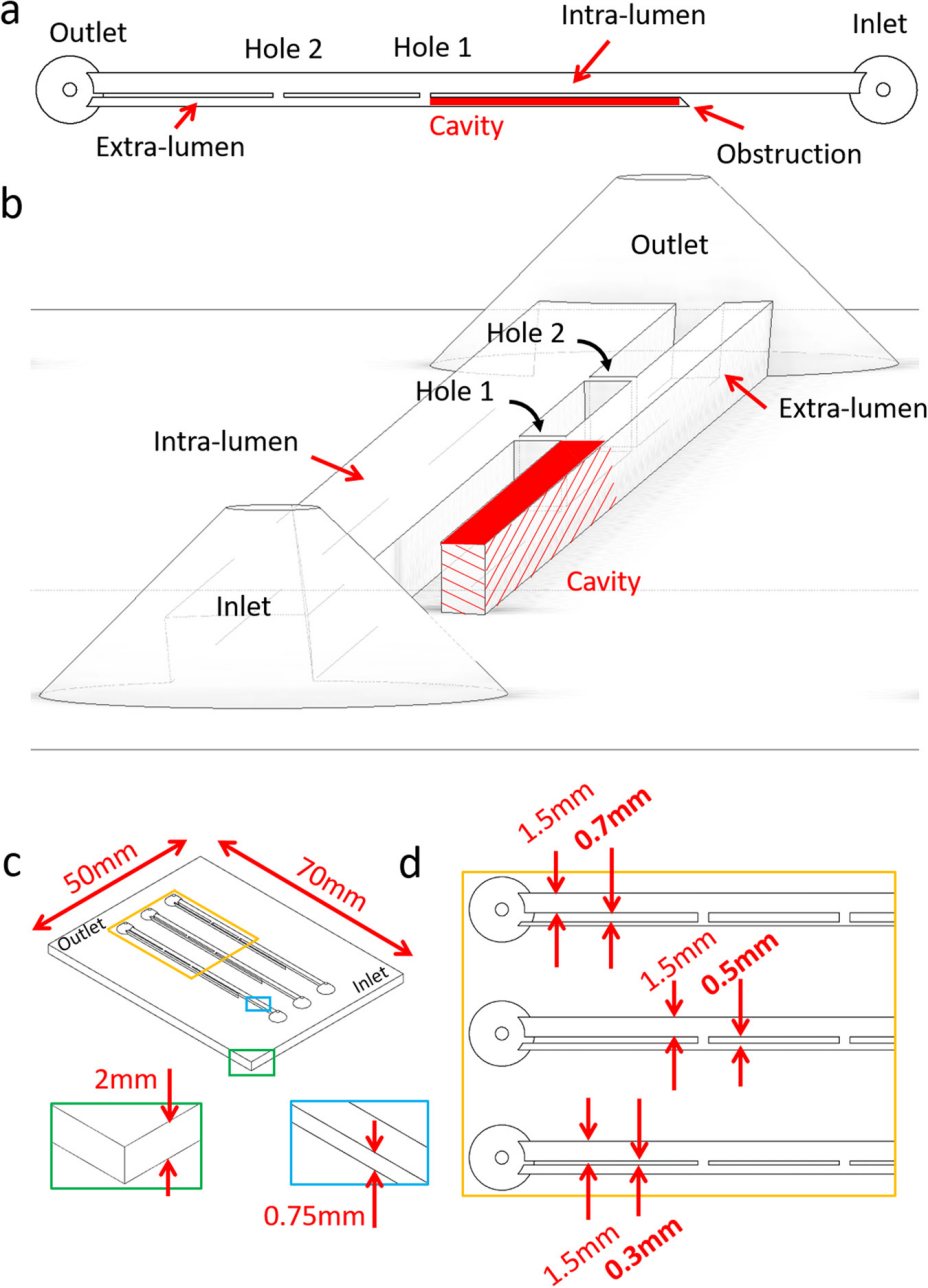
Top view (a) and 3D isometric view (b) of the model, illustrating a typical architecture and its relevant hydrodynamic regions (i.e., Inlet, Outlet, Hole 1, Hole 2, Cavity, and Intra/Extra-lumen). (c) Isometric view of the full SoC device. The blue and green boxes provide a zoomed-in view of the channel height and the SoC base, respectively. The yellow box provides a zoomed-in view of the proximal region of the SoC model. This is illustrated in (d) and comprises models with different septum thickness (in bold) of 0.3 mm, 0.5 mm, and 0.7 mm, and a constant intra-luminal width of 1.5 mm. Channels in this model have a rectangular cross section.

Briefly, the model included two main flow channels mimicking intra- and extra-luminal compartments of a stented ureter system, respectively [Fig. 2(a)]. These two channels were separated by a septum, modeling the stent wall. The septum contained two side-channels connecting intra- and extra-luminal compartments, and was designed to model side-holes of a ureteric stent (as in Fig. 1). In addition, two reservoirs were positioned at the inlet and outlet of the model. It should be noted that the channel replicating the extra-luminal compartment was not directly connected to the inlet reservoir [Fig. 2(a)]. The dead end of this channel was designed to model the hydrodynamic cavity generated by a complete obstruction of the ureter lumen,^26^ where the cavity is in a position distal to the occlusion [see region labeled in red, in both Figs. 1 and 2(a)]. Physical dimensions of the geometrical features of the stent (i.e., side-hole size, distance between adjacent side-holes, stent inner diameter, and thickness) were taken from a commercially available double-J stent (model Universa^®^, Cook^®^ Medical, USA), and are reported in Fig. 2(c).

The internal diameter of the physiological human ureter varies along its length, ranging from approximately 5.3 mm to 1.9 mm and has a mean value of approximately 3.4 mm.^27^ In this study, SoC models were designed to replicate a complete occlusion of the upper ureteric tract (see Fig. 1), where the ureter diameter at the site of occlusion was set to 3.0 mm. Notably, a study by Moon *et al*. on 246 patients with unilateral ureteral stone has revealed that 37% of obstructions lodged in the upper ureter.^28^ The mean length of the human ureter is in the range 175-286 mm,^27,29^ while the length of the ureteric compartment in this study was set to 60 mm. Thus, SoC models only replicate a segment of the stented ureter system, containing characteristic flow domains such as “active” and “inactive” side-holes, and the cavity formed by complete ureteric obstructions (Fig. 1). A single SoC platform included three replicas of the model [see Fig. 2(b)] to increase the experimental throughput.

### B. Methodological rationale

The present investigation builds on the existence of an inverse correlation between WSS and deposition-rate of encrusting particles in the stented and occluded ureter, as determined in our previous study,^24^ which suggests that a reduction in stent encrustation could be achieved by increasing the WSS levels. This could potentially result from either an increase in ureteric flow rate or through alterations of the stent design to locally perturb the WSS field, with the latter approach likely to be more practicable in a clinical scenario.

Here, we employed SoC models to identify stent architectures capable of reducing encrustation over regions that are particularly susceptible to it (i.e., side-holes and the occluded cavity). Specifically, the effects of varying the stent thickness and side-hole shape were investigated. Computational fluid dynamic (CFD) simulations and experiments were performed to determine the spatial distribution of WSS and encrusting deposits, respectively, within multiple SoC architectures.

The study was articulated into three main phases. In the *first phase*, the effect of varying the stent wall thickness was evaluated, by using SoC models with three different septum thicknesses, namely:

(a) 0.5 mm, which is a value commonly used in the clinic and is herein referred to as “standard” or “0.5S”;
(b) 0.3 mm, corresponding to a thinner stent wall that is also used clinically^19^ and is herein referred to as “0.3S”;
(c) 0.7 mm, corresponding to a thicker stent wall compared to the standard ones^30^ and is herein referred to as “0.7S.”

The intra-luminal width was kept constant in this phase of the study. This first series of experiments and numerical simulations allowed (i) to identify a stent wall thickness resulting in the lowest rate of encrustation and (ii) to further assess whether WSS (obtained from CFD simulations) correlated with the amount of deposited encrustation (obtained experimentally). Moreover, point (ii) provided confidence that the CFD model could be used as a reliable tool for researching hydrodynamically optimised stent geometries. Therefore, in the *second phase* of the study, CFD simulations were employed to identify a side-hole shape that reduced the extent of fluid stagnation (i.e., regions with low-WSS) and consequently decreased the overall encrustation rate. Specifically, streamlined side-hole architectures with different vertex angles were investigated. Finally, in the *third phase* of the study, a SoC model combining the best performing stent wall thickness and side-hole shape was designed, and its performance was compared with the standard SoC design (0.5S) using both CFD simulations and experiments.

### C. Numerical simulation of the flow field

Computational fluid dynamic simulations were performed to resolve the flow field within SoC models. Attention was given to the WSS distribution at regions of the model that were found to be susceptible to encrustation, such as side-holes of the stent and the occluded cavity.^24^ Fluid properties and boundary conditions in the numerical model were defined to replicate the experimental ones, as reported in our previous study.^24^ A constant volumetric flow rate of 1 ml/min was imposed at the SoC inlet, coherently with previous *in silico*^26,31^ and *in vitro*^32^ models. This flow rate is within the physiological range for adults (0.8-1.5 ml/min), as reported in a study on five males and three females in the age range 21-33 years^33^ and a recent *in vitro-in vivo* extrapolation conducted by Matsuzaki *et al.*^34^ The inlet Reynolds number (calculated in the intraluminal compartment of the stent) is defined as Re_in_ = *(ρVD_h_)/μ* and, in this study, was equal to ∼15, where *ρ* is the fluid density (997.044 kg/m^3^), *V* is the mean fluid velocity in the inlet channel (1.48 cm/s), *D_h_* is the hydraulic diameter of the inlet channel, and *μ* is the dynamic viscosity of the fluid (0.001 Pa s). Notably, values of mean fluid velocity and Reynolds number in this study are comparable to those reported in previous models of the stented urinary tract.^29,35^ Atmospheric pressure was imposed at the outlet boundary.

Initially, a design of the SoC was built using Autodesk^®^ Inventor Pro 2018 (Autodesk, USA). Subsequently, three-dimensional (3D) drawings (in STEP format) were imported in ICEM CFD 18.1 (Ansys Inc., USA), where the fluidic domain was discretised in finite volumes of tetrahedral shape. When defining the numerical mesh, attention was devoted to the spatial resolution of the wall shear stress (WSS) field over the bottom surface (or bed) of the SoC models. A mesh volume edge length of 0.05 mm was selected as a compromise between computational cost and solution robustness, and this was assessed through a grid convergence study (see the supplementary material). Different SoC designs were simulated in this study, and the number of mesh volumes ranged between 12 515 349 and 13 975 780. The fluid dynamic field was computed in Fluent^®^ 18.1 (Ansys Inc., USA) by solving for laminar, incompressible, and steady-state mass and momentum conservation equations, as detailed in our previous study.^24^

### D. Fabrication of SoC devices

SoC devices were manufactured using the micromilling-replica moulding (μMi-REM) technique, following the protocol described by Carugo *et al.*^36^ Briefly, a positive mould was first created by casting epoxy adhesive resin (yellow dual cartridge, RS Components Ltd., UK, at 1:1 weight ratio between components) over a negative PMMA mould. Liquid polydimethylsiloxane (PDMS, Sylgard^®^ 184, Dow Corning Corporation, USA, curing agent:monomer ratio of 1:10 by weight) was then poured over the positive mould and allowed to solidify (at 65 °C overnight). Subsequently, the PDMS layer containing the SoC channel architecture was permanently sealed to a 1 mm thick microscopic glass slide (70 mm × 50 mm, Sigma-Aldrich^®^, USA), by treatment with oxygen plasma (plasma asher TePla 300, PVA-TePla^®^, Germany). Upon completion of the manufacturing process, devices were employed without any chemical treatment of their inner surfaces.

### E. Experimental quantification of encrustation

**FIG. 3.**
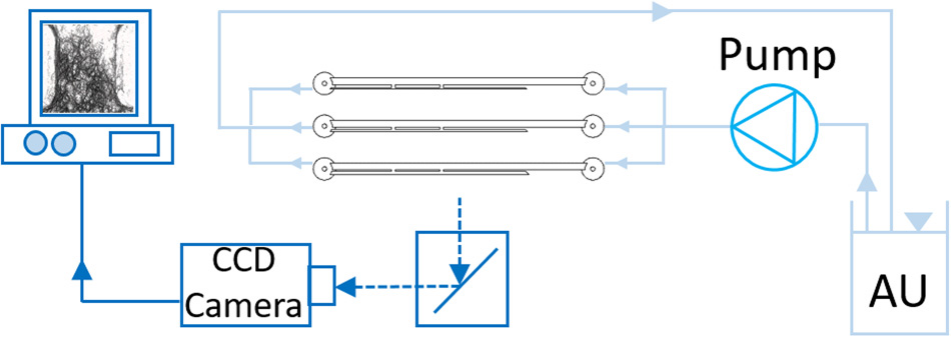
Experimental setup employed to quantify deposition of encrusting particles within SoC models. It included a reservoir containing artificial urine (AU), a peristaltic pump, the SoC device placed horizontally on a microscope stage, a CCD camera for capturing microscope images, and a PC for image storage and post-processing.

Deposition of encrusting particles within SoCs was quantified using the experimental setup shown in Fig. 3. It consisted of two main units: (i) the *flow unit*, to deliver a urine surrogate within SoCs, and (ii) the *optical unit* for capturing the deposition of encrusting particles *in situ*.

The preparation of the surrogate fluid (or artificial urine, AU) was carried out following a protocol designed by Brooks and Keevil,^37^ with minor modifications (as reported in Mosayyebi *et al.*^24^). All chemical constituents were purchased from Sigma Aldrich^®^ (UK). The AU solution was kept at 37 °C (under continuous stirring) and pH of 6.8, during experimentation. Tubing (PTFE, Cole-Parmer^®^, UK) connected the device to a reservoir containing the AU solution and to a peristaltic pump (Minipuls3, Gilson^®^, UK) used to deliver AU through the SoC devices at a physiologically relevant flow rate of 1 ml/min (as used for the numerical simulations). The experiment was run for 90 min, which was deemed sufficient to detect the initial stages of crystals' deposition and thus identify domains that are likely to act as initial anchoring sites for encrustation. The SoC device was placed horizontally on the stage of an optical microscope. An inverted microscope (Wilovert 30, Helmut Hund GmbH, Germany) and a CCD camera GXCAM-HICHROMESII (GT-Vision^®^, UK) with exposure time set to 1 ms were employed to acquire images of encrusting deposits within SoCs (at 5× magnification). Images were acquired every 15 min and were then processed using ImageJ software (NIH, USA) to quantify the time evolution of encrustation at selected locations (i.e., Hole 1, Hole 2, and Cavity). The image processing followed a procedure described previously^24^ and quantified the percentage area covered by encrusting deposits over specific regions of interest within the models (also referred to as “mean coverage area”). Three independent experimental repeats were carried out, and three images were taken at each location and time point (for each repeat).

### F. Statistical analysis

The experimental data are presented as mean ± standard deviation. Differences between particle deposition in Hole 1, Hole 2, and Cavity were evaluated using unpaired Welch's t-test. The correlation between the parameters of time and the mean coverage area was evaluated by conducting a linear regression analysis. The significance level (Alpha) was set to 0.05 (i.e., differences were considered to be statistically significant for P < 0.05). Additionally, a Kolmogorov Smirnov test (KS-test) was employed to evaluate the significance of the effect of flow rate (in Hole 1, Hole 2, and Cavity) for a fixed SoC design and to statistically evaluate the significance of the effect of stent design (in Hole 1, Hole 2, and Cavity) for a fixed flow rate. Rstudio (RStudio^®^, USA) and XLSTAT (XLSTAT^®^, USA) software were employed to perform the statistical analyses.

## III. RESULTS AND DISCUSSION

### A. Effects of changing the stent wall thickness

#### 1. Effect of stent wall thickness on the WSS field

**FIG. 4.**
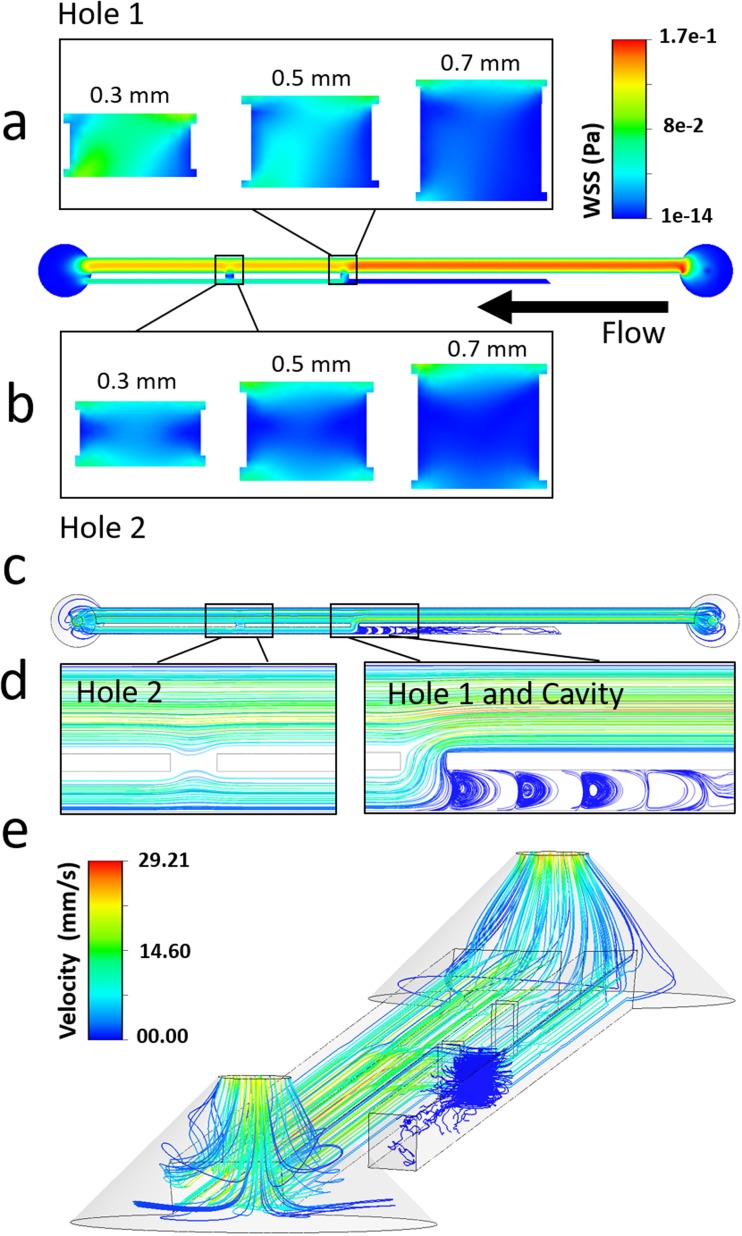
Contours of WSS magnitude at (a) Hole 1 and (b) Hole 2, taken at the bottom-wall (or bed) of the SoC model (i.e., in the *z*-direction), for stent wall thicknesses of 0.3 mm, 0.5 mm, and 0.7 mm. The black arrow indicates the main flow direction. (c–e) Fluid pathlines in the SoC model, colored by fluid velocity magnitude. Pathlines are reported for (c) the all model (top view), (d) the regions close to Hole 1, Hole 2, and Cavity (top view), and (e) the all model (isometric view, with a lower pathline density). Results are reported for a SoC model with a wall thickness of 0.3 mm.

Figure 4 shows the WSS spatial distribution over the bottom-wall (or bed) in both Hole 1 and Hole 2, for the three different stent wall thicknesses investigated (see Fig. 2). When comparing different side-holes, Hole 2 (i.e., the more distal to the obstruction) was associated with a lower mean WSS (0.021 Pa, 0.015 Pa, and 0.010 Pa, for wall thicknesses of 0.3 mm, 0.5 mm, and 0.7 mm, respectively) because of the partially stagnant flow in this region [see dark blue areas in Fig. 4(b)]. This is also confirmed by observing the fluid pathlines in proximity to Hole 2 [see Fig. 4(d)], showing the absence of inter-compartmental flow exchange at this side-hole.

On the other hand, in Hole 1 (i.e., located just after the occlusion), inter-compartmental flow exchange occurred because of the presence of the obstruction [see pathlines in Figs. 4(c) and 4(d)]. This enforced a significant increase in mean WSS, which was equal to 0.054 Pa, 0.030 Pa, and 0.012 Pa for wall thicknesses of 0.3 mm, 0.5 mm, and 0.7 mm, respectively. In Cavity, the formation of low-velocity counter-rotating eddies was observed, as illustrated in Figs. 4(c)–4(e).

From the moment that the postulated function of side-holes in ureteric stents is to promote fluid drainage, we refer to Hole 1 and Hole 2 as “active” and “inactive” side-holes, respectively.

Importantly, numerical results illustrated in Fig. 4 are in agreement with a previous numerical study by Tong *et al*.,^17^ showing that the first side-hole post-obstruction is characterised by the largest proportion of inter-compartmental fluid exchange.

**FIG. 5.**
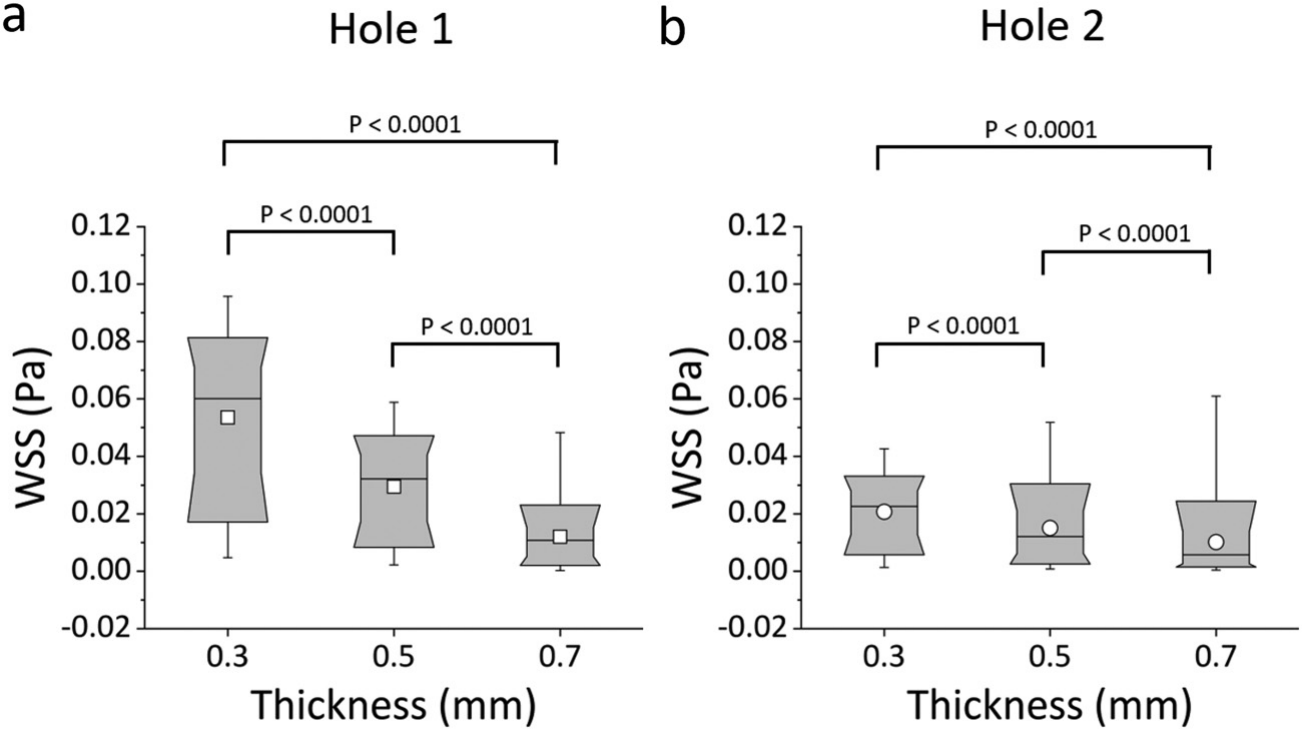
Box plots showing the distribution of WSS (in Pa) over (a) Hole 1 and (b) Hole 2 of the SoC models. Values were calculated numerically at a flow rate of 1 ml/min, for different stent wall thicknesses of 0.3 mm, 0.5 mm, and 0.7 mm. Box plots show the distribution of the WSS over the ranges of 10%, 25%, 50%, 75%, and 90% of the total distribution. The empty squares correspond to the mean WSS values. P-values are reported for statistically significant comparisons, determined through a Kolmogorov Smirnov test.

Box plots in Figs. 5(a) and 5(b) show the distribution of WSS values over the bottom-wall of Hole 1 and Hole 2, respectively. Results confirm that there was a very significant reduction in mean WSS at both Hole 1 and Hole 2, when increasing the stent wall thickness; i.e., 0.7 mm *vs.* 0.5 mm (P < 0.0001), 0.7 mm *vs.* 0.3 mm (P < 0.0001), and 0.5 mm *vs.* 0.3 mm (P < 0.0001). The “active” Hole 1 was affected by changes in the wall thickness to a greater extent compared to the “inactive” Hole 2. This is likely due to the absence of flow exchange through the more distal side-hole, reducing its sensitivity to changes in the global architecture of the stent. At the greatest stent wall thickness investigated (0.7 mm), only a small difference in mean WSS between Hole 1 and Hole 2 was observed, suggesting that stents with a thicker wall may experience limited drainage through both “active” and “inactive” side-holes.

#### 2. Effect of stent wall thickness on the deposition of encrusting particles

**FIG. 6.**
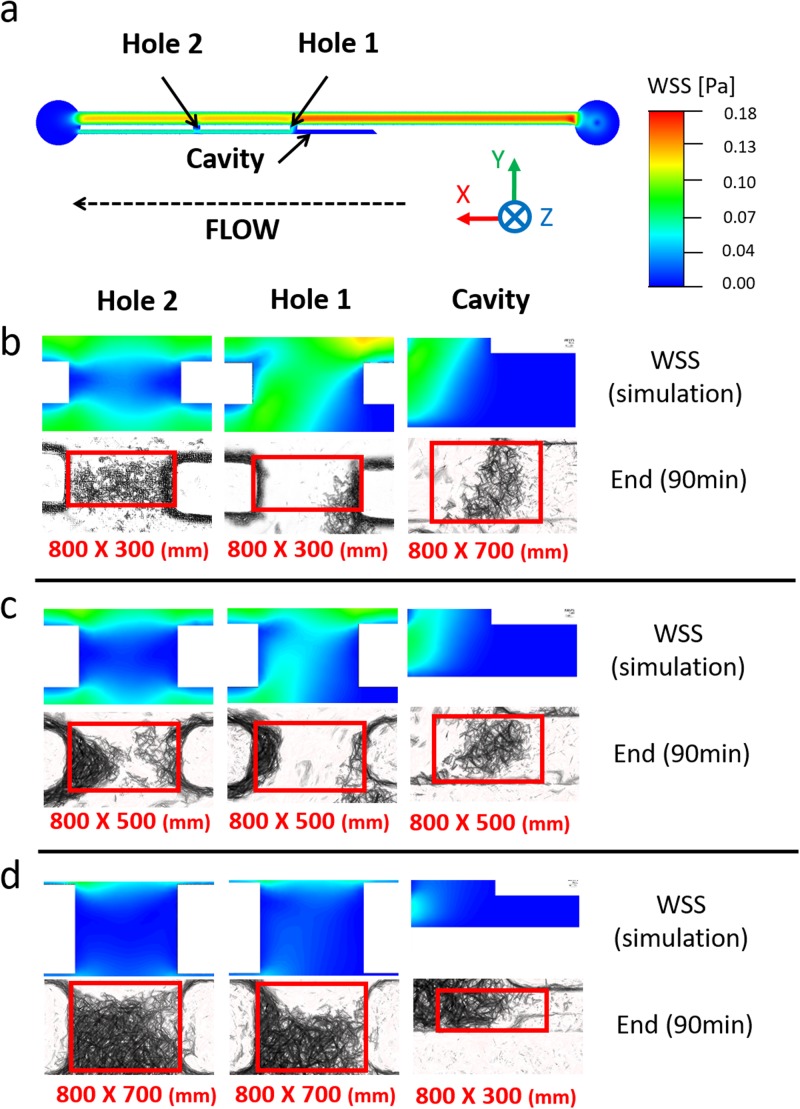
(a) Contour of WSS magnitude at the bottom plane of the SoC model, computed numerically. WSS contours are reported at three hydrodynamic regions of interest within the model (Hole 1, Hole 2, and Cavity), together with the corresponding microscope images showing accumulation of encrusting deposits. Experiments were run at an inlet flow rate of 1 ml/min, using SoC devices with wall thickness of (b) 0.3 mm, (c) 0.5 mm, and (d) 0.7 mm. Images were taken at 90 min from the beginning of the experiment (n = 3). Red boxes (with their size underneath them, reported as “width × height” in mm) correspond to the regions over which the percentage area covered by encrustation was calculated.

Figure 6 shows the spatial distribution of WSS over the bottom-wall of the SoC model, compared to the corresponding experimental images showing accumulation of encrusting crystals. The comparison is made over three regions of interest (i.e., Hole 1, Hole 2, and Cavity) and for different stent wall thicknesses (i.e., 0.3 mm, 0.5 mm, and 0.7 mm). An inverse correlation between the magnitude of WSS acting over the stent wall and the amount of encrusting deposits can be observed, for most of the experimental conditions investigated. Low levels of encrustation occurred in regions characterised by high WSS, and vice versa. Moreover, deposition of encrusting particles in side-holes was initiated at the lateral edges of the hole, where the WSS was lower. This finding may explain the presence of an encircling crystallization at side-holes of double-J stents retrieved from patients.^38^ Results also confirm that the occluded cavity is a region prone to the deposition and growth of encrusting particles, and this applies to all stent thicknesses investigated. This observation is in agreement with our previous findings using both microfluidic-based models^24^ and a full-scale artificial model of the ureter,^26^ which revealed the presence of laminar vortices in the cavity formed by a ureteric obstruction (see Fig. 4) and their role in promoting trapping and deposition of encrustation.

**FIG. 7.**
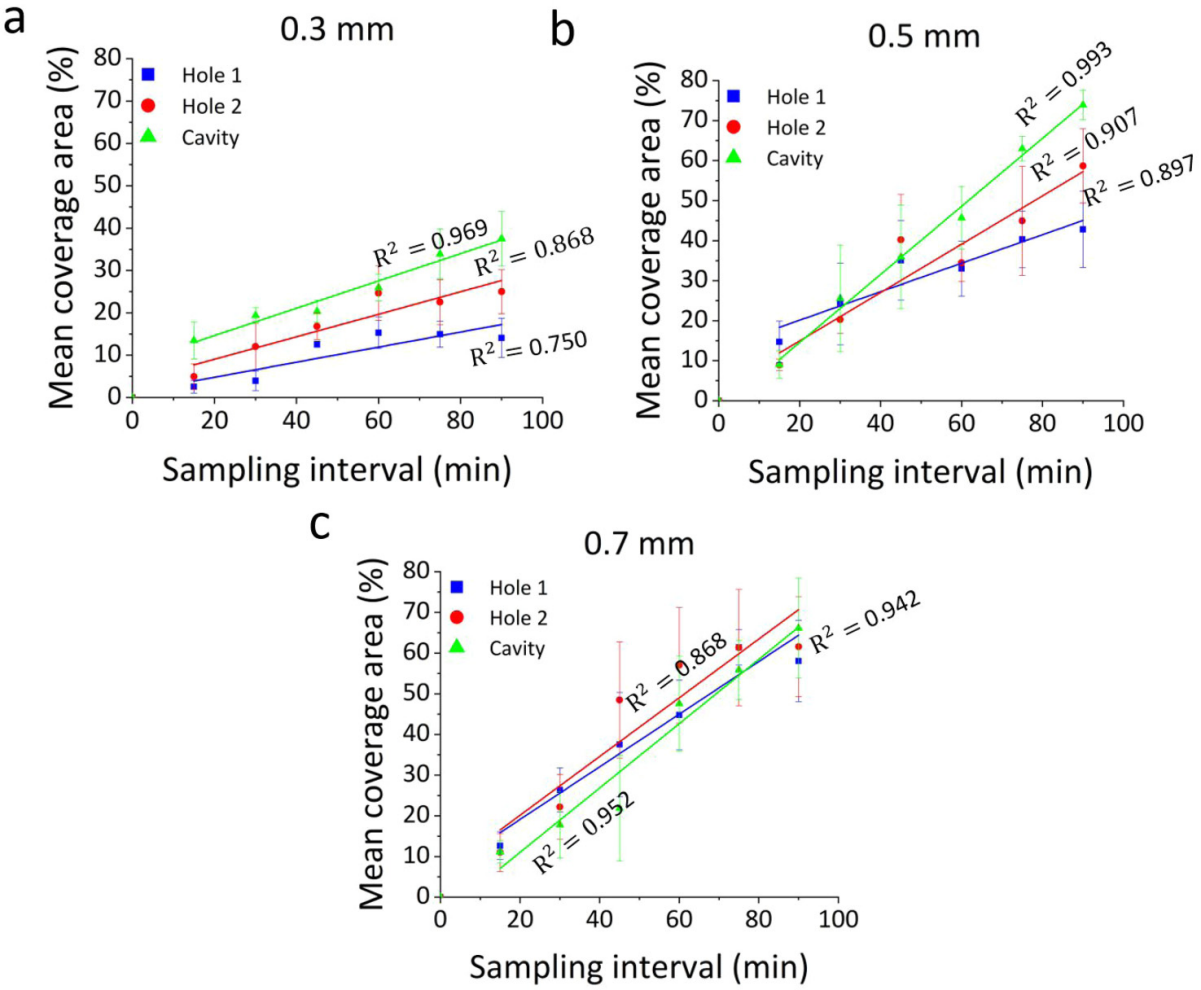
Time evolution of the percentage area covered by encrusting deposits, at specific regions of interest within the models (Hole 1, Hole 2, and Cavity). Results are reported for inlet flow rate of 1 ml/min, and at three wall thicknesses of (a) 0.3 mm, (b) 0.5 mm, and (c) 0.7 mm. Data are expressed as mean value ± standard deviation of three independent repeats. Linear regression functions (for the time interval: 15 min–90 min) are plotted, together with the corresponding R^2^ values.

Figure 7 shows a quantitative analysis of the level of encrustation over time, within side-holes and the occluded cavity, for the different wall thicknesses investigated. Values were obtained from image analysis, by measuring the percentage area covered by encrusting deposits. The red boxes in Fig. 6 show the spatial regions over which the percentage area covered by encrustation was calculated.

Results showed that a wall thickness of 0.3 mm is associated with the lowest deposition of encrustation, after 90 min of continuous artificial urine flow. Hole 1, Hole 2, and Cavity had mean coverage area of 14.1% ± 4.6%, 25.0% ± 5.2%, and 37.5% ± 6.4%, respectively. At a wall thickness of 0.5 mm, the mean coverage area was 42.8% ± 9.5% (Hole 1), 58.7% ± 9.3% (Hole 2), and 73.9% ± 3.7% (Cavity), while at a wall thickness of 0.7 mm, it was 58.1% ± 10% (Hole 1), 61.5% ± 12.3% (Hole 2), and 66% ± 12.3 (Cavity). Hole 2 and Cavity had statistically lower mean coverage area at a thickness of 0.3 mm in comparison to a thickness of 0.5 mm (Hole 2: P = 0.0476; Cavity: P = 0.0140). Meanwhile, the mean coverage area at Hole 1 for a wall thickness of 0.3 mm was significantly lower compared to a wall thickness of 0.7 mm (P = 0.0315). Interestingly, at a wall thickness of 0.7 mm, the percentage area covered by encrustation was comparable across the different regions investigated (mean values were 58.1% ± 10.0%, 61.5% ± 12.3%, and 66.1% ± 12.3% for Hole 1, Hole 2, and Cavity, respectively). This suggests that, at higher stent wall thicknesses (>0.5 mm), both “inactive” and “active” side-holes are susceptible to comparable levels of particle deposition, as they are both subject to low-WSS levels (as shown in Fig. 5).

Results from this first phase of the study indicate that reducing the stent wall thickness promotes inter-compartmental urine drainage, which in turn leads to higher WSS levels at side-holes of the stent. These hydrodynamic effects contribute to reducing encrustation rates at both “active” and “inactive” side-holes, and within the occluded cavity. A stent wall thickness of 0.3 mm was found to be associated with the lowest encrustation rate, among the different wall thicknesses investigated. A further reduction of the stent thickness could be potentially evaluated, but its clinical translation may be hindered by the low resistance to mechanical stress during insertion.

Furthermore, in line with our previous investigation,^24^ an inverse correlation between the magnitude of WSS and the amount of encrusting deposits over the stent surface was observed. This provides confidence in using numerical models to perform further hydrodynamic optimisation of the stent architecture, as discussed in Secs. III B and III C.

### B. Effects of changing the side-hole shape

Results discussed above confirm that both the occluded cavity and “inactive” side-holes of the stent are prone to the deposition of encrustation across a range of clinically relevant stent wall thicknesses, due to the low-WSS levels in these regions. Notably, previous studies suggested that a large proportion of side-holes in ureteric stents made an insignificant contribution to inter-compartmental flow exchange and may thus be obstructed by crystalline deposits.^17,39^ These findings were further corroborated by recent numerical studies by Kim *et al*.,^40,41^ where the ureter architecture was reconstructed from two-dimensional axial computed tomography (CT) data collected from 19 men. It should however be noted that these prior studies were conducted on ureter models with a fixed cross-sectional area, which did not replicate the compliant nature of the ureter wall nor the presence of physiological ureteric constrictions. These previous findings however suggest that side-hole activation may occur only in close proximity to either pathological or physiological constrictions of the ureter lumen.

Therefore, in this phase of the study, we investigated the effect of changing the side-hole design on the encrustation rate in these functional regions of the stent. CFD simulations were initially employed to determine the spatial distribution and magnitude of WSS within SoC models characterised by different side-hole shapes. Based on the findings discussed above, a stent wall thickness of 0.3 mm was selected in this second phase of the study, being the most effective in reducing encrustation rate at side-holes.

#### 1. Effect of side-hole shape on the WSS field

**FIG. 8.**
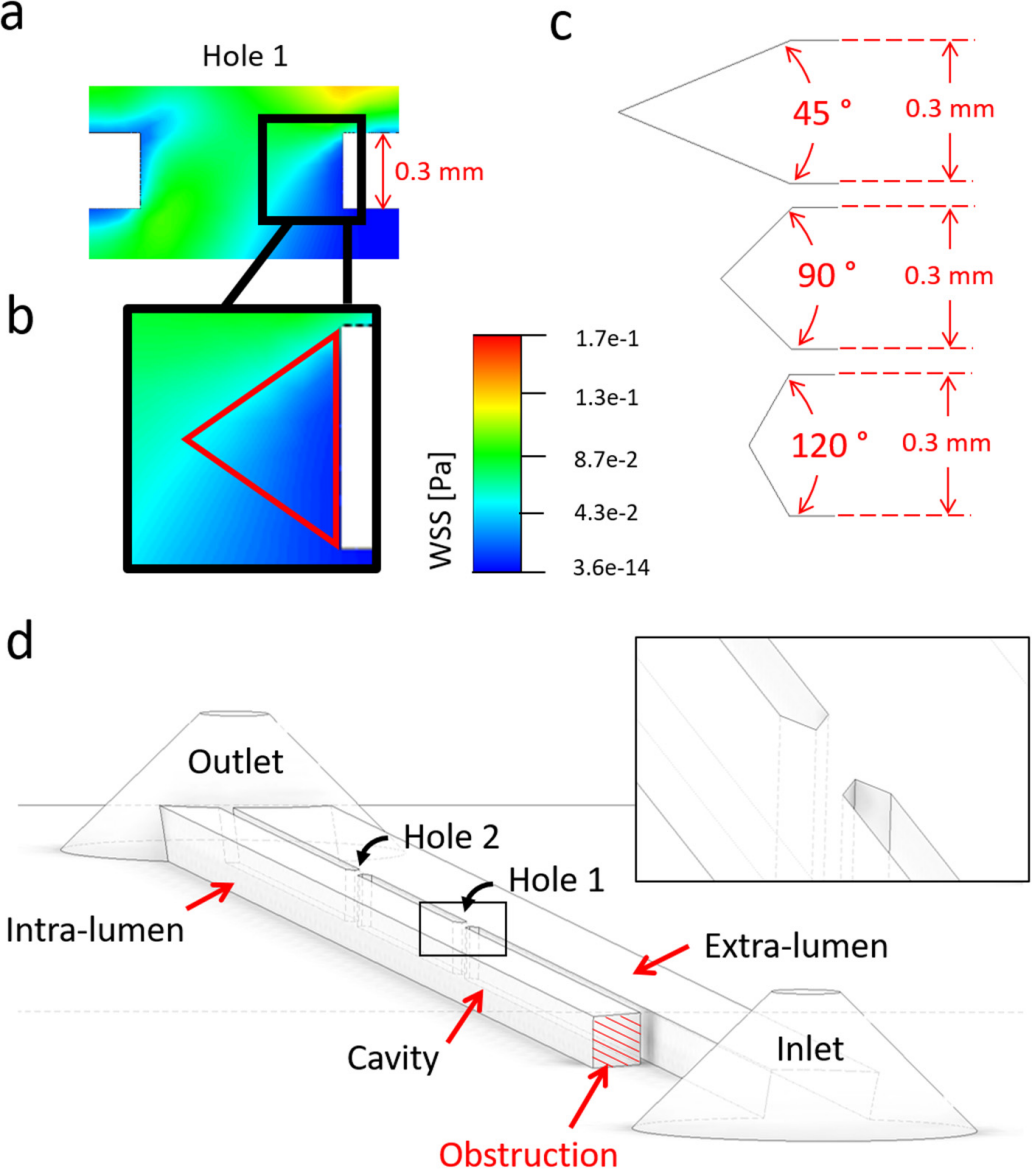
(a) Contours of WSS magnitude (in Pa) in Hole 1 of the SoC model, computed numerically at an inlet flow rate of 1 ml/min, for a stent wall thickness of 0.3 mm. The inset in (b) shows a zoomed-in view of the right wall of the side-hole, with low-WSS values shown in dark blue color. A red isosceles triangle illustrates a hypothetical side-hole shape. (c) Schematic of different streamlined side-hole shapes investigated in this study, each characterised by a different vertex angle of either 45°, 90° or 120°. (d) 3D isometric view of a SoC model (wall thickness: 0.3 mm, vertex angle: 45°) with streamlined side-hole shape. A zoomed-in view of Hole 1 is shown in the inset.

Numerical simulations using a standard side-hole shape (see Fig. 4) revealed that regions suffering from low WSS (i.e., WSS < 0.02 Pa) at side-holes presented an approximately triangular shape. It was therefore hypothesized that “streamlined” side-holes with a triangular architecture could lead to a significant increase in the average WSS, particularly in the more critical “inactive” side-holes of the stent. Side-holes were thus shaped as isosceles triangles, with three different vertex angles (45°, 90°, and 120°), as shown in Fig. 8. For consistency, the width of each hole (i.e., distance between vertex tips in triangular walls) was kept constant and equal to the width of the standard hole shape (0.8 mm). Moreover, the lateral distance between two adjacent side-holes was kept to a constant value of 10 mm.

**FIG. 9.**
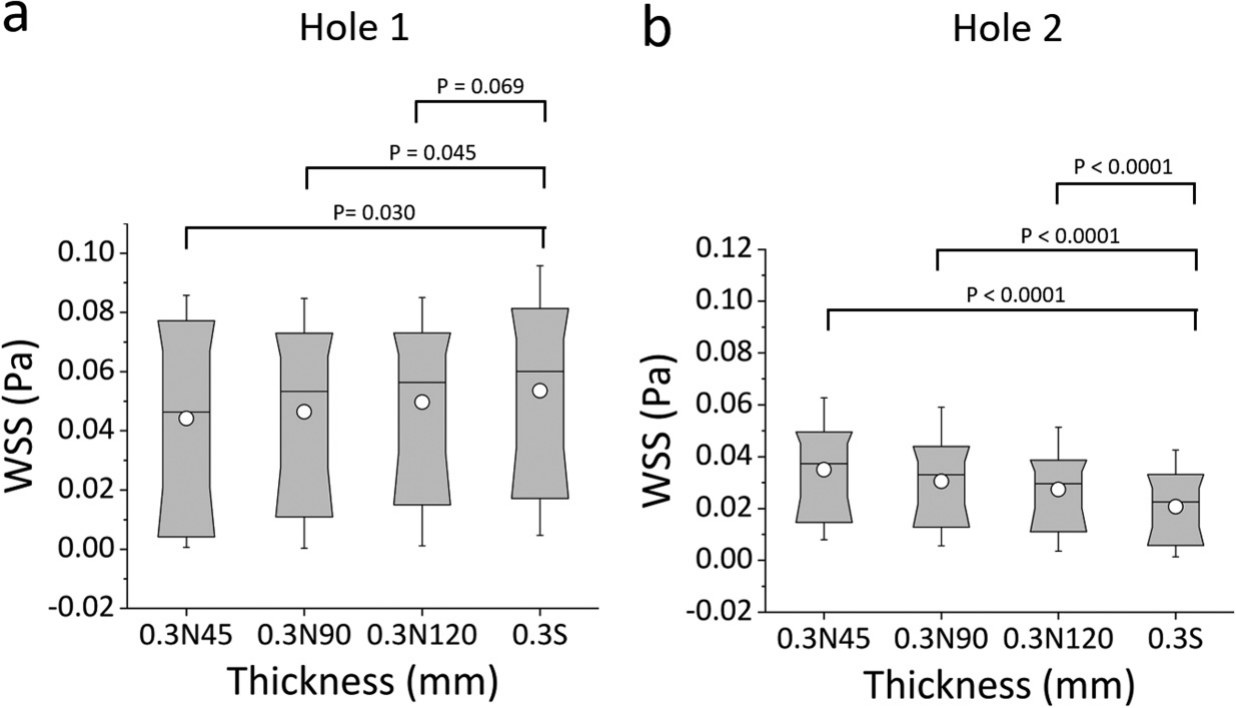
Box plots showing the distribution of WSS values (in Pa) over (a) Hole 1 and (b) Hole 2 of SoC models. Values were calculated numerically at an inlet flow rate of 1 ml/min, for a standard side-hole shape (rectangular with straight walls) and triangular (isosceles) side-holes with vertex angles of 45° (0.3S45), 90° (0.3S90), and 120° (0.3S120). Box plots show the distribution of WSS over the ranges of 10%, 25%, 50%, 75%, and 90% of the total distribution. The statistical significance was determined using the KS-test, and p-values are reported above the box plots for statistically significant comparisons.

Box plots in Fig. 9 show the distribution of WSS values calculated numerically, for the standard side-hole shape (0.3S) and the three aforementioned triangular shapes having vertex angles of 45° (0.3N45), 90° (0.3N90), and 120° (0.3N120). Results show that the triangular geometries provided a clear benefit—in terms of increasing the magnitude of WSS—only for Hole 2. The standard side-hole shape (0.3S) provided the lowest median WSS value (0.02226 Pa). When streamlined side-hole shapes were employed, median WSS values at Hole 2 were equal to 0.0373 Pa, 0.0331 Pa, and 0.0296 Pa for 0.3N45, 0.3N90, and 0.3N120, respectively, and differences between side-hole designs were very statistically significant (0.3S *vs.* 0.3N45: P < 0.0001; 0.3S *vs.* 0.3N90: P < 0.0001; 0.3S *vs.* 0.3N120: P < 0.0001).

In Hole 1, the effect of a triangular geometry was slightly detrimental, as the distribution of WSS shifted toward lower values (median WSS was equal to 0.005 Pa, 0.060 Pa, 0.053 Pa, and 0.059 Pa for 0.3N45, 0.3S, 0.3N90, and 0.3N120, respectively). It should however be noted that shape optimization is more critical for Hole 2, as “inactive” side-holes are significantly more numerous in ureteric stents and are more prone to encrustation compared to “active” side-holes, as reported in previous investigations.^39^

**FIG. 10.**
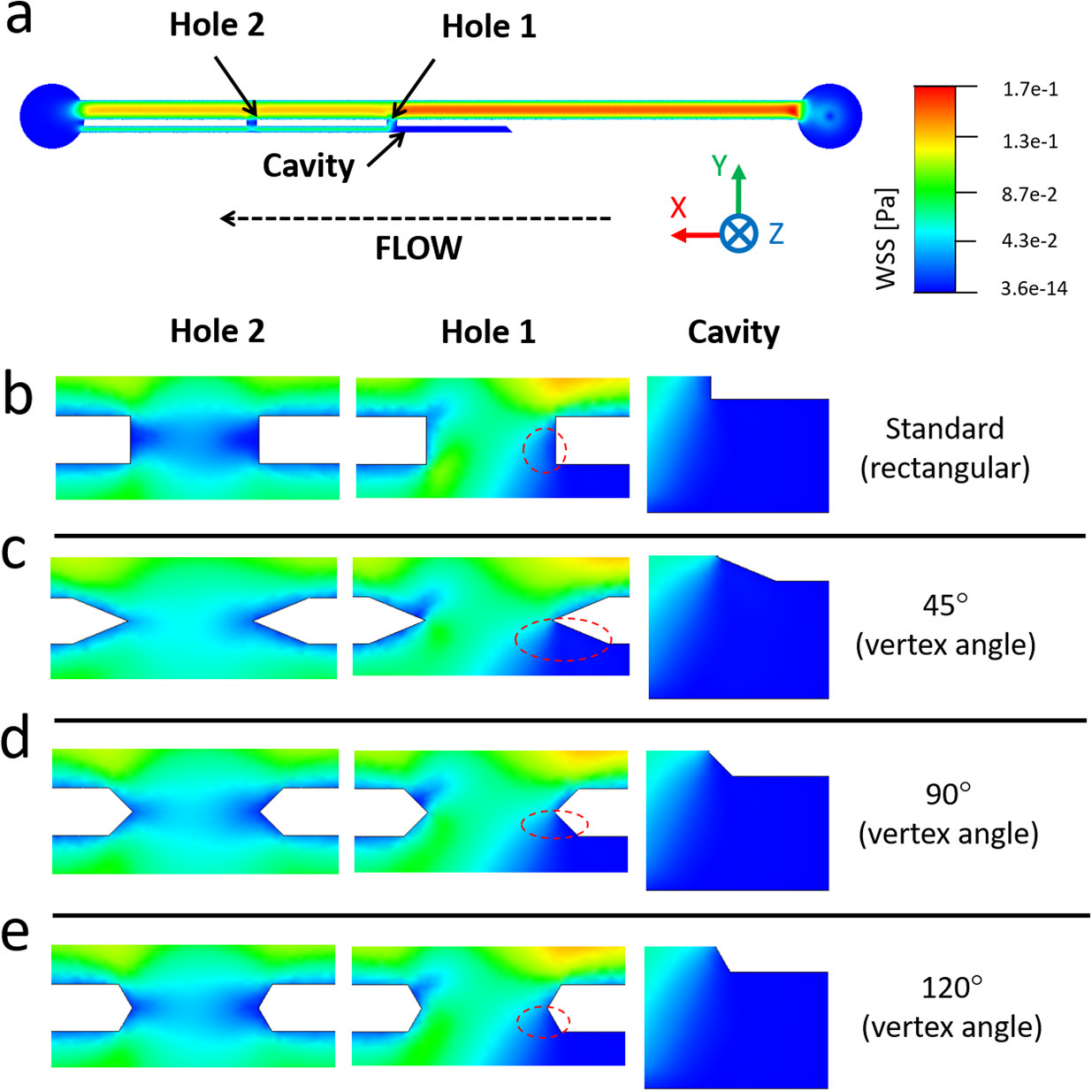
(a) Contours of WSS magnitude at the bottom plane of the SoC model, computed numerically. (b) Contours of WSS are reported at three hydrodynamic regions within the model (Hole 1, Hole 2, and Cavity), at an inlet flow rate of 1 ml/min and vertex angles of (c) 45°, (d) 90°, and (e) 120°. Red dashed circular/oval boxes indicate stagnant regions with low-WSS, located in proximity to Hole 1 and facing the extra-luminal compartment of the model.

This is further illustrated in Fig. 10, where contours of WSS are reported for all investigated side-hole geometries. In Hole 1, inter-compartmental fluid exchange was guaranteed regardless of the side-hole shape, and WSS values were overall relatively high (mean WSS > 0.04 Pa). The streamlined side-hole with the 45° vertex angle was the best performing, as it resulted in the most significant increase in WSS levels. Therefore, 0.3N45 was selected as the most promising side-hole architecture and subject to further investigations.

### C. Combined effects of stent wall thickness and side-hole shape

#### 1. Characterisation of the WSS field: Optimised vs. standard stent architecture

**FIG. 11.**
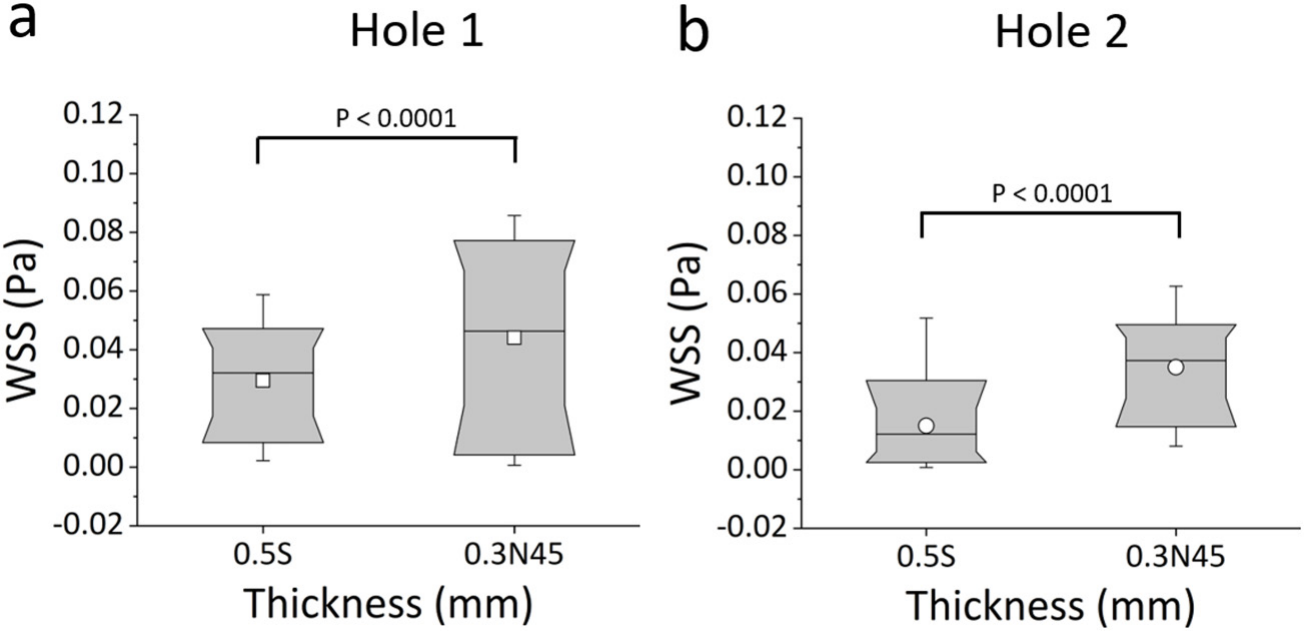
Box plots showing the distribution of WSS values (in Pa) over (a) Hole 1 and (b) Hole 2 of the SoC models. Values were computed numerically at an inlet flow rate of 1 ml/min, for both the standard stent design (referred to as 0.5S) and the new design (referred to as 0.3N45). Box plots show the distribution of WSS over the ranges of 10%, 25%, 50%, 75%, and 90% of the total distribution. Empty squares correspond to the mean values. The statistical significance was determined using the KS-test, and P-values are reported above the box plots for statistically relevant comparisons.

Box plots in Fig. 11 show the distribution of WSS values at “active” (Hole 1) and “inactive” (Hole 2) side-holes of both the original stent with standard thickness (0.5S) and the new design combining optimised wall thickness and side-hole shape (0.3N45). The results demonstrate a statistically significant decrease in mean WSS from 0.044 Pa (for 0.3N45) to 0.029 Pa (for 0.5S) in Hole 1 (P < 0.0001), and from 0.035 Pa (for 0.3N45) to 0.015 Pa (for 0.5S) in Hole 2 (P < 0.0001), corresponding to a percentage relative change of 49.8% and 133.4%, respectively.

#### 2. The optimised stent architecture reduces deposition of encrusting particles

**FIG. 12.**
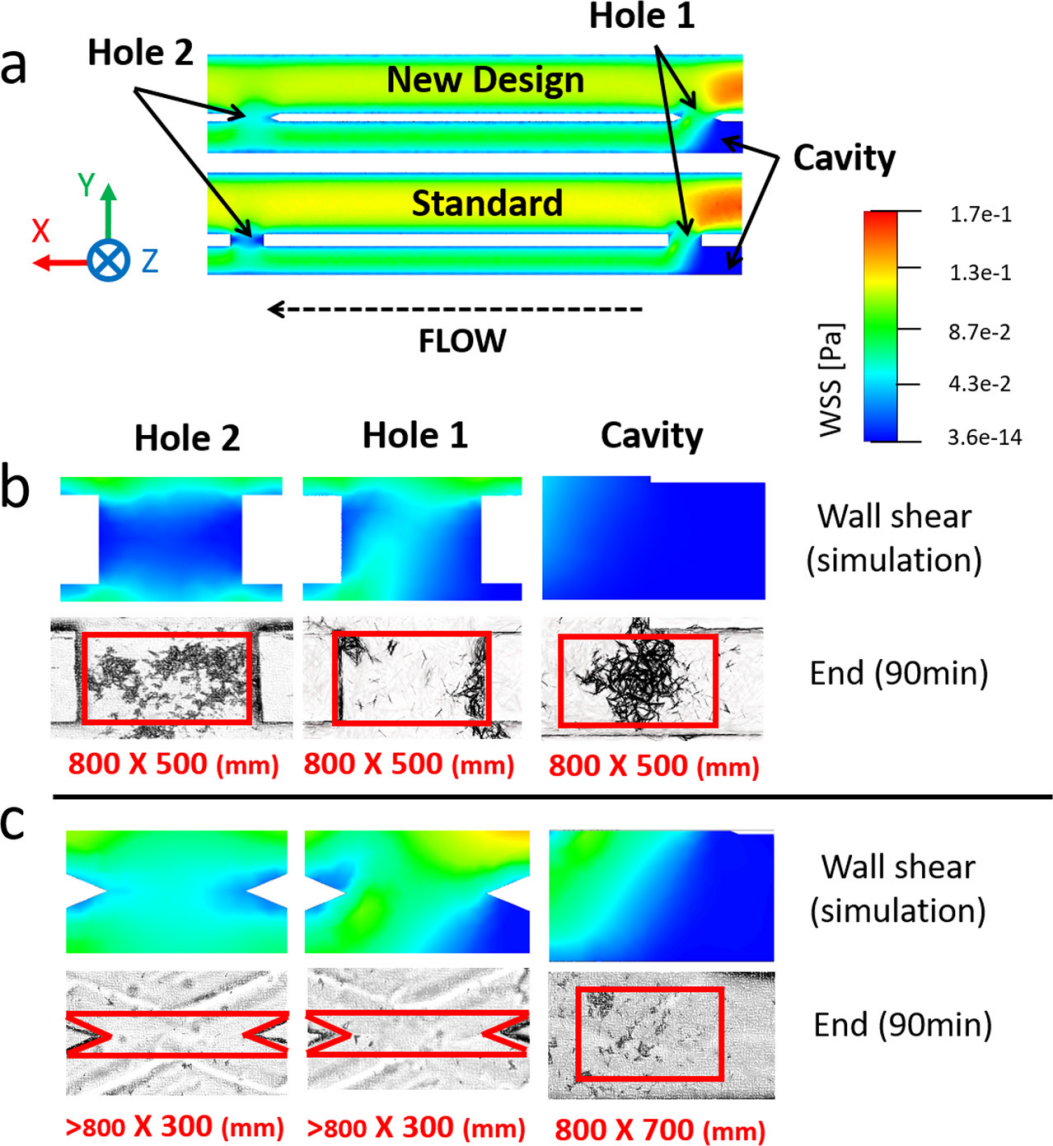
(a) Contours of WSS magnitude at the bottom plane of the SoC model, computed numerically for both standard design and optimised design. (b) and (c) Contours of WSS at three hydrodynamic regions within the model (Hole 1, Hole 2, and Cavity) and corresponding microscope images showing encrusting deposits, at an inlet flow rate of 1 ml/min and for both (b) standard shape (referred to as 0.5S) and (c) optimised design (referred to as 0.3N45). Images were taken at 90 min from the beginning of the experiment (n = 3). Red boxes (with their size reported underneath, as “width × height” in mm) correspond to the regions of interest over which the percentage area covered by encrustation was calculated.

Figure 12 shows the spatial WSS distribution over the bottom-wall of the model and experimental images of encrustation, both taken at three regions of interest (Hole 1, Hole 2, and Cavity). Experiments again demonstrated an inverse correlation between the simulated WSS field and the amount of encrusting deposits.

**FIG. 13.**
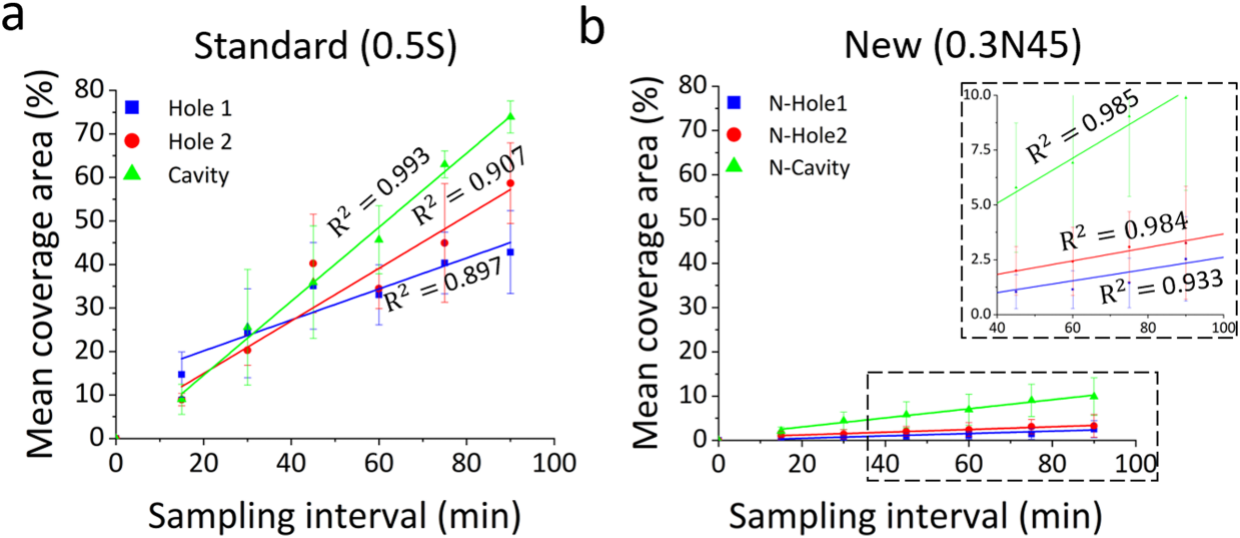
Time evolution of the percentage area covered by encrustation, at specific regions of interest within the models (Hole 1, Hole 2, and Cavity). Results are reported for inlet flow rate of 1 ml/min, and for both (a) the standard design (referred to as 0.5S) and (b) the optimised design (referred to as 0.3N45). Experimental data are expressed as the mean value ± standard deviation of three independent repeats. Linear regression models (for the time interval 15 min–90 min) are plotted, together with the corresponding R^2^ values.

This significant improvement is further illustrated in Fig. 13 which shows the time evolution of the percentage area covered by encrustation in the regions of interest (highlighted in red in Fig. 12). The optimised stent design was characterized by significantly lower levels of encrustation compared to the standard design, in any region of interest. The experimental data have also been analyzed using a linear regression model, where the slope of the regression function represents the encrustation rate (in min^−1^). Results show that both side-holes and the cavity region experienced a drastic reduction of mean coverage area in 0.3N45 compared to the standard design (0.5S) (Hole 1: R^2^ = 0.897, P = 0.004, encrustation rate = 0.36 min^−1^; Hole 2: R^2^ = 0.907, P = 0.003, encrustation rate = 0.6 min^−1^; and Cavity: R^2^ = 0.993, P < 0.0001, encrustation rate = 0.85 min^−1^). In particular, in the 0.3N45 design, Hole 1, Hole 2, and Cavity showed a % relative reduction of encrustation of 94.1%, 94.4%, and 86.6%, respectively (Hole 1: R^2^ = 0.933, P = 0.002, encrustation rate = 0.02 min^−1^; Hole 2: R^2^ = 0.984, P < 0.0001, encrustation rate = 0.03 min^−1^; and Cavity: R^2^ = 0.985, P < 0.0001, encrustation rate = 0.01 min^−1^).

## IV. LIMITATIONS AND FUTURE PERSPECTIVES

The model employed in this study presents some limitations, as described below.

(a) Channels have a rectangular cross section which does not mimic the physiological stented ureter. It however enables high-resolution bright field optical imaging. Future work will focus on developing three-dimensional models of the stented ureter and more accurately replicating the physiological cross-sectional shape of the ureter (both *in-vitro* and *ex-vivo*). This may however require integration with alternative imaging modalities (i.e., based on X-ray or fluorescence) for detecting encrustation *in situ*.
(b) The SoC model replicates only a limited segment of the stented ureter system, as it aims at recapitulating key hydrodynamic domains within a compact and microscope-compatible platform. Ureteric stents typically contain numerous side-holes; thus, a more comprehensive understanding of their function would require the development of full-scale models of the ureteric tract. However, a previous full-scale numerical study by Tong *et al.*^17^ revealed that the first hole post-obstruction was subject to the largest proportion of inter-compartmental fluid exchange, while the other side-holes remained largely “inactive.” These findings were corroborated by recent numerical studies by Kim *et al*.,^40,41^ where the ureter architecture was reconstructed from CT data. These previous investigations suggest that the majority of side-holes in a stent may be characterised by comparable levels of WSS. However, the presence of localised constrictions in the physiological ureter will likely affect inter-compartmental flow distribution and would thus merit further investigations, encompassing *in vitro* and *ex vivo* experiments.
(c) Previous studies have shown that the physiological urodynamics is impaired in the stented and occluded ureter. In particular, the presence of a stent has been shown to cause a significant reduction in peristaltic activity.^42^ Therefore, we assumed that stationary walls and continuous flow would be appropriate modeling conditions in this study. However, a more comprehensive investigation of stent encrustation may also require modeling of multiple urine discharges to replicate the priming-voiding cycle of the bladder.
(d) The present study focused on the effect of different geometrical characteristics of the stent on the initiation of particle deposition. The long-term progression of encrustation is however likely to be affected by the surface properties of the model; thus, future work may focus on utilising SoC models to evaluate the effect of surface characteristics or functionalisation methods.
  Moreover, the shape of encrusting deposits will in turn influence the WSS distribution in the stented ureter and potentially the progression of the encrustation process in the long-term. Therefore, alternative imaging modalities may be employed in the future to obtain a three-dimensional reconstruction of the occlusion over time, which can be used to further inform the fluid dynamic modeling.
(e) Encrustation of urinary stents involves several physical, chemical, and microbiological processes, each with different temporal dynamics.^43^ In this study, we utilised a urine surrogate to isolate the effect of multiple geometrical stent parameters on the deposition of encrusting crystals. However, we anticipate that differences in the composition of urine between patients may affect the rate of encrustation in stents. Future work will thus investigate the effect of changing the chemical and biological composition of the working fluid, on the formation and growth of encrusting crystals.

## V. CONCLUSION

A stent-on-chip microfluidic model was employed as a screening platform to investigate the effect of changing architectural design features of a ureteric stent on the deposition of encrusting particles. A main focus of this study was placed on encrustation of side-holes of the stent, as they have been previously identified as primary anchoring sites for encrusting particles and given their important functional role in maintaining urine drainage. The effects of changing the thickness of the stent wall (in the range 0.3-0.7 mm) and the shape of side-holes (standard *vs.* streamlined) were evaluated. An inverse correlation between the magnitude of WSS and the deposition rate of encrusting particles was observed, for all geometrical configurations investigated. This confirms our previous findings and expands beyond commercial stent geometries. Reducing the stent wall thickness increased the WSS magnitude in both “active” and “inactive” side-holes of the stent, with a 0.3 mm thick stent characterised by the lowest amount of encrustation. Introducing a streamlined side-hole architecture further contributed to reducing encrustation rates, particularly at “inactive” side-holes. Overall, combining both optimal stent wall thickness (0.3 mm) and side-hole shape (streamlined, with 45° vertex angle) resulted in a large increase in WSS within both “active” and “inactive” side-holes and the cavity, causing a significant reduction in the deposition of encrustation in these regions (i.e., relative reduction of 94.1% in Hole 1, 94.4% in Hole 2, and 86.6% in Cavity).

To the best of the authors' knowledge, the present manuscript describes the first study utilising a microfluidic-based approach to investigate the effect of design-mediated changes to the WSS distribution on the deposition of encrustation in ureteric stents. Using this approach, streamlined side-hole shapes for usage in ureteric stents have been proposed and evaluated for the first time. Findings from this research may inform academic and industrial laboratories in their search for more effective and safer stenting technologies. Notably, the proposed fluid dynamic-based approach against deposition of encrusting particles in ureteric stents could be applied to different bulk materials or surface coatings, making it potentially suitable for integration with different clinical or industrial approaches. Future studies are however required in order to validate the performance of the optimised stent design under more physiologically relevant conditions. On-going research is currently being performed in our laboratories using 3D numerical and experimental models, which replicate the macroscopic urinary flow dynamics. Studies using pre-clinical models are also being performed to investigate the tolerability of the proposed side-hole design *in-vivo*.

In addition, a similar microfluidic-based design approach against encrustation could be applied to the optimisation of other medical devices suffering from similar complications, such as biliary or pancreatic stents.^44–46^

## SUPPLEMENTARY MATERIAL

See supplementary material for the result of the numerical mesh convergence study.
